# Sequential autohydrolysis-organosolv pretreatment for cellulose derivative production

**DOI:** 10.1007/s00449-026-03425-4

**Published:** 2026-09-19

**Authors:** Fabrícia Vieira, Hortência E. P. Santana, Osiris Ashton Vital Brazil, Isabelly Pereira Silva, Daniel Pereira Silva, Denise Santos Ruzene

**Affiliations:** 1 RENORBIO – Northeastern Biotechnology Network, Federal University of Sergipe, São Cristóvão, SE 49107-230 Brazil; 2 Plural Cooperative of Work, Consulting, Research, and Services, São Paulo, SP 04013-040 Brazil; 3 CCET – Center for Exact Sciences and Technology, Federal University of Sergipe, Rodovia Marechal Rondon, s/n - Jardim Rosa Elze, SE 49107-230 São Cristóvão, Brazil; 4 PROBIO – Graduate Program in Biotechnology, Federal University of Sergipe, São Cristóvão, SE 49107-230 Brazil; 5 PPGPI – Graduate Program in Intellectual Property Science, Federal University of Sergipe, São Cristóvão, SE 49107-230 Brazil; 6 CISAS – Center for Research and Development in Agrifood Systems and Sustainability, Instituto Politécnico de Viana do Castelo, 4900-347 Viana do Castelo, Portugal

**Keywords:** Aautohydrolysis, Organosolv, Coconut mesocarp, Lignin, Carboxymethyl cellulose

## Abstract

Pretreatment is a crucial stage in obtaining cellulose pulp from coconut residue. It helps in extracting lignin and hemicellulose, increasing the efficiency of the further process. This study proposed and developed a sequential pretreatment combining autohydrolysis, organosolv, and chemical bleaching to maximize the efficiency of extractive, hemicelluloses, and lignin removal. The aim was to explore and enhance the extraction of high-quality cellulose pulp derived from coconut residues for the production of carboxymethyl cellulose (CMC). Chemical and structural analysis was performed to confirm the efficiency of the proposed process. Chemical analysis confirmed the removal of lignin and hemicellulose, resulting in an enhancement of cellulose content from 40.01% to 78.92% FT-IR analyses confirmed modifications in the chemical structure of cellulose, while TGA revealed a significant mass loss in the cellulose-specific temperature range, demonstrating the efficient removal of other components. SEM images, in turn, provided microscopic visualizations that proved the successful preparation of CMC. The process resulted in a CMC yield of 30.75%, demonstrating its effectiveness in preparing cellulosic pulp suitable for CMC production, which stands out as a promising method for using coconut fibers in industry.

**Graphical abstract:**

**Figure:**
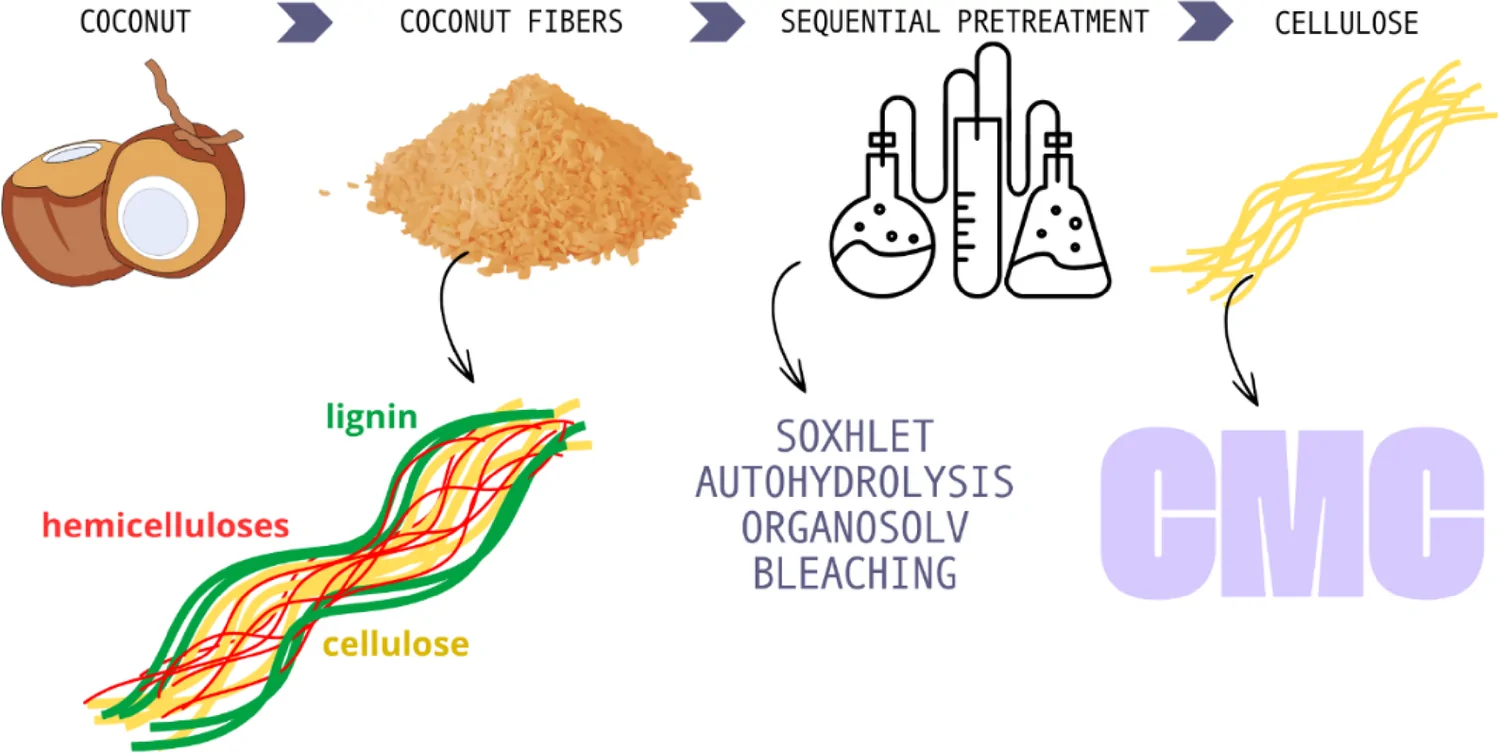

**Supplementary Information:**

The online version contains supplementary material available at https://doi.org/10.1007/s00449-026-03425-4.

## Highlights

- Efficient sequential pretreatment for removing extractives, hemicellulose, and lignin.
- Lignin removal confirmed by chemical analyses, Kappa number, and Klason lignin.
- FTIR, TGA, and SEM analyses show increasing cellulose in coconut fibers.
- Efficient pretreatment sequence for producing CMC from coconut fibers.
- Valorization of coconut residues for bioproducts via sequential pretreatment.

**Supplementary Information:**

The online version contains supplementary material available at https://doi.org/10.1007/s00449-026-03425-4.

## Introduction

Advancing biorefineries is crucial for sustainable development and a circular economy, enabling the valorization of plant-based feedstocks into high-value bioproducts [1]. Cellulose, as the dominant lignocellulosic component, is central within these processes. However, biorefineries face persistent operational challenges, including volatile and uneven biomass supply, high operating costs, and low conversion efficiencies when processing lignocellulosic components as a whole [2].

In terms of sustainable resource utilization, coconut residues stand out as a promising renewable lignocellulosic resource, driven by their abundance, versatility, and widespread consumption of the fruit. Including parts such as the fibrous mesocarp (coconut fibers, CF), hard shell, and defatted coconut pulp, these residues are continuously supplied due to the global demand for coconuts and their derivatives, like milk, flour, oil, and sugar [3].

However, lignocellulosic biomass has a complex structure that remains difficult to decompose even after pretreatment. Its recalcitrance makes it challenging to efficiently depolymerize lignin-carbohydrate complexes (LCC), often leading to low lignin extraction and poor-quality cellulosic pulp [4]. Because no single universal method exists for LCC depolymerization, extensive research has explored various pretreatment conditions and combinations to improve lignocellulosic biomass fractionation, particularly by removing lignin and hemicelluloses to increase cellulose accessibility.

Gundupalli et al. [5] investigated the effect of pretreatments with different ionic liquids on the solubilization of hemicelluloses and lignin in CF. The glucose yield from the CF pretreated with pyrrolidin-1-ium hydrogen sulfate and triethylammonium hydrogen sulfate increased by 4.12 and 4.73 times, respectively. Meanwhile, the delignification was 15.76% and 18.27%, respectively. Campos et al. [6] submitted coconut fibers (CF) to the autohydrolysis (AH) pretreatment at 201 °C for 10 min and observed a reduction in the extractive content and an improvement in cellulose content compared to hemicelluloses from 3.34 to 4.53 g/g. It was also observed that the ratio of cellulose to lignin reduced from 1.59 to 1.29 g/g. Their results indicate convergence on the effectiveness of AH pretreatment in removing the hemicelluloses fraction, thereby enhancing the solubilization of components and increasing the accessibility of cellulose.

Avelino et al. [7] evaluated the OG delignification of coconut shells using different acid catalysts. The highest lignin yield (56.6%) was obtained using an aqueous acetic acid solution with 2.0% AlCl_3_, which also produced high-purity (83%), low-molecular-weight lignin under mild conditions. Vieira et al. [8] investigated various experimental conditions for AH, alkaline extraction (AE), and OG on CF. They identified optimal combinations for each method for lignin extraction: AH at 195 °C for 20 min, AE using 0.5 M NaOH for 2 h, and OG with NaOH as a catalyst for 2 h. The concentrations of lignin extracted were 1.80 g/L, 9.71 g/L, and 99.9 g/L, respectively. To evaluate the blend of coffee husk, beans-caupi husk, and cacao husk, Morais et al. [9] conducted acid pretreatment followed by enzymatic hydrolysis, using the obtained sugar to produce bacterial cellulose. The hydrolysate showed 54.45% (m/m) glucose and 87% cellulose digestibility, indicating the efficacy of pretreatment.

To improve lignocellulosic biomass conversion efficiency, multiple pretreatment methods may be combined to integrate the strengths of different techniques. Based on the literature, sequential treatment, which has not been discussed in depth [10], is shown to be innovative and effective in depolymerizing biomass and obtaining high-quality lignocellulosic fractions. Meighan et al. [11] applied sequential pretreatment (AH-OG) to sugarcane bagasse, followed by NaOH washing to remove re-precipitated lignin. The method achieved efficient delignification, yielding solids rich in cellulose for enzymatic hydrolysis and 2G bioethanol production. AH at 175.8 °C for 49 min produced pulp with 28.91% lignin, 63.53% cellulose, and 9.29% hemicelluloses. After OG (210 °C, 120 min), delignification reached 84.63%, reducing the pulp lignin content to 7.08%. Ruiz et al. [12] investigated the delignification of wheat straw using a sequential AH-OG process. They used AH at 180 °C for 30 min, solubilizing 44% of xylan and recovering 78% of sugars as xylooligosaccharides. The enriched solid fraction, consisting of 63.7% glucan and 7.55% xylan, underwent an OG process with 40% ethanol and 0.1% NaOH at 180 °C for 20 min, yielding 3.25 g/100mL of extracted lignin. The cellulose fractions presented crystallinity indices of 21.32% (native), 55.17% (post AH), and 53.59% (post OG).

Although the sequential pretreatment AH-OG strategy has demonstrated efficiency in delignification for different biomass feedstocks, its specific application in coconut biomass has not yet been explored. Thus, assessing the AH-OH strategy for coconut residues is relevant to determining its effectiveness in extracting high-quality lignocellulosic fractions, especially cellulose-rich pulp, for the synthesis of cellulose derivatives such as methyl cellulose, hydroxymethyl cellulose, and carboxymethyl cellulose (CMC) [13]. AH pretreatment is an environmentally friendly approach that disrupts the lignocellulosic structure using only water as a reagent and time and temperature as parameters [14]. The OG process promotes delignification using organic solvents such as ethanol, making it cost-effective and sustainable for obtaining high-quality lignin and cellulose [15].

CMC is an industrially important cellulose derivative, mostly because of its physicochemical characteristics, which include solubility in water, insolubility in ethanol, a pH range of 6 to 8.5, and a degree of substitution varying from 0.2 to 1.5 [16]. Conventionally, CMC is produced using wood or cotton linters, depending on the required purity [17]. The process generally involves isolating and purifying cellulose with hydrochloric acid and an alkaline solution, followed by reaction with chloroacetic acid to convert it into CMC [18]. Although these conventional feedstocks provide the required cellulose quality for CMC, researchers are interested in using alternative lignocellulosic residues.

Accordingly, researchers have explored different pretreatments to obtain CMC from other lignocellulosic biomass. Vedia et al. [19] utilized ionic liquid pretreatment on sugarcane bagasse, while Olawuyi et al. [20] produced CMC from okra stalks using deep eutectic solvents. However, to the best of our knowledge, no previous study has investigated the sequential application of AH-OG pretreatment to coconut residue with the aim of producing a cellulose-rich pulp for CMC production.

To address gaps in the biomass valorization literature, this study investigates a sequential AH-OG pretreatment designed to enhance cellulose accessibility for posterior conversion into CMC. Unlike individual methods, the proposed sequences integrate AH for the removal of hemicelluloses with OG to facilitate further delignification and enrichment of cellulose. The approach aims to establish a more sustainable and cost-effective pathway for the valorization of abundant lignocellulosic biomass into value-added cellulose derivatives. Consequently, this research advances the field by assessing the efficacy of AH-OH for fractionating coconut biomass while also demonstrating its applicability for the production of high-value products such as CMC.

## Materials and methods

### Samples processing

Coconut fiber (CF) was collected from mature coconuts sourced from an industrial facility in northeast Brazil. CF was washed, dried, and manually cut to 2 cm, then homogenized. Samples *in natura* were used for chemical and structural analysis, and some underwent the sequential pretreatment (Figure S1).

### Soxhlet extraction

Before pretreatment, CF samples were initially submitted to Soxhlet extraction to remove extractives. This step is conducted to eliminate impurities and other non-lignocellulosic components from the sample surface. The process involved recirculating 99.8% PA ethanol as a solvent for 4 h at the boiling point, with 6 cycles per hour. The CF samples were then dried in an oven at 50 °C for 24 h.

### Autohydrolysis-organosolv sequential pretreatment

For the autohydrolysis (AH) step, extractive-free CF was treated with distilled water (1:25 g/g biomass: water) in a stainless steel reactor submerged in a silicone oil bath at 195 °C for 20 min. After the reaction, the reactor was cooled in an ice bath for 10 min, and the mixture was vacuum-filtered to separate solids from the filtrate.

Subsequently, the solid fraction obtained in AH was subjected to organosolv (OG) delignification. The samples were transferred to a stainless-steel reactor and treated with an ethanol-water (1:1) solution in the presence of NaOH (10% dry weight) as a basic catalyst, keeping the solid-to-liquid ratio at 1:25. The system was placed in a silicon oil bath preheated to 185 °C and maintained for 2 h. Following the reaction time, the reactor was cooled in an ice bath for 10 min, and the liquid phase was separated from the solid through vacuum filtration.

The experimental conditions employed for AH and OG pretreatments were based on previous work by Vieira et al. [8]. After each pretreatment, the filtered sample was collected to analyze soluble (SL) and insoluble lignin (IL). The AH solid fraction was washed with distilled water, while post OG was washed with a 1:1 ethanol: water solution, at a solid-to-liquid ratio of 1:20, followed by drying in an oven at 55 °C for 24 h. A portion of each solid sample was used for structural and chemical characterization, while the remaining part was kept for the subsequent step (OG or chemical bleaching).

### Chemical bleaching

The pretreatment of AH-OG CF samples was followed by chemical bleaching to remove residual lignin from the pulp. These samples were placed in Erlenmeyer flasks with distilled water and subjected to mechanical agitation until a homogeneous material was obtained, after which they were transferred to a thermostatic bath to reach thermal equilibrium at 70 ± 1 °C. Next, acetic acid and sodium chlorite were added [21]. After 60 min, the samples were cooled in an ice bath to 10 °C, filtered, washed with distilled water until a neutral pH was attained, and then dried in an oven at 50 °C [22]. Samples followed for the chemical characterization and CMC synthesis.

### CMC synthesis

Bleached pulp was mixed with ethanol and distilled water, and then a NaOH solution was added dropwise over 30 min while maintaining mechanical agitation at 15 ± 1 °C. Next, a 50% monochloroacetic acid and ethanol solution was added dropwise over 30 min under continuous agitation at 20 ± 1 °C. After addition, the temperature was raised to 60 °C, and the mixture was maintained under agitation for 3 h before filtration. The solid phase was suspended in 70% methanol and neutralized with 90% acetic acid. The suspension was vacuum filtered, and the precipitate was washed repeatedly with ethanol and methanol and dried in an oven at 60 ± 1 °C [23]. The mass of CMC weight gain was determined by Eq. 1.

1 $$\:\%CMC\:weight\:gain=\left(\frac{{CMC}_{\left(g\right)}}{{Bleached\:pulp}_{\left(g\right)}}\right)*100$$

### Chemical characterization of pretreated samples

#### Pulp and lignin yield

The yield of pretreatment processes was determined by considering the relation between the final mass of each sample obtained post-treatment and the initial mass, as outlined in Eq. 2.

2 $$\:\%Process\:yield=\left(\frac{{final\:mass}_{\left(g\right)}}{{initial\:mass}_{\left(g\right)}}\right)*100$$

Concerning lignin, the yields were determined based on the mass of lignin extracted during the process and the mass used, as described in Eq. 3.

3 $$\:\%lignin\:yield=\left(\frac{{extracted\:lignin}_{\left(g\right)}}{{MC\:mass}_{\left(g\right)}}\right)*100\:\:\:\:\:$$

#### Total lignin determination

The total lignin concentration (TL) was calculated by summing both IL and SL. The methodology for determining IL followed the procedure of Ismail et al. [24]. In this process, 40 mL aliquots of liquor from each pretreatment step (AH and OG) were treated with sulfuric acid until the pH was adjusted to 2, reducing lignin solubility and causing it to precipitate.

After acidification, the solution underwent centrifugation, and the supernatant was used to determine SL. The precipitate was washed with distilled water, centrifuged to eliminate any residual acid, and then dried in an oven. SL concentrations were measured using UV-visible spectroscopy at 205 nm (spectrophotometer Bel Photonics spectrophotometers, Model UV-M51, Valinhos/SP, Brazil). The wavelength was selected based on previous studies, which identified 205 nm as effective because lignin contains numerous chromophores that absorb strongly in this UV region [25].

#### Determination of Kappa number and Klason lingin

The kappa number was calculated to determine the amount of lignin remaining in the solid samples after each pretreatment step, as well as in the lignin present in the untreated biomass. Experiments were conducted according to the TAPPI T 236 om-99 [26], which establishes a correlation between the Kappa number and the percentage of Klason lignin in pulp (Eq. 4).

4 $$\:\%Klason\:lignin=Kappa\:EquationNumber*0.13$$

#### Determination of holocellulose, α-cellulose and hemicelluloses

The holocellulose and α-cellulose contents were determined according to procedures also applied by Vieira et al. [18]. For holocellulose, samples were treated with acetic acid and sodium chloride in an Erlenmeyer flask and placed in a water bah at 70 °C for 4 h. The holocellulose samples were washed with acetone and dried at room temperature; α-cellulose was determined by treating holocellulose samples with 17.5% NaOH in a water bath at 20 °C, applying NaOH in two steps with varying quantities and durations (2 mol/L). Then, the hemicelluloses content was determined by the difference between holocellulose content and α-cellulose .

### Structural characterization

#### Fourier transform infrared spectroscopy (FT-IR)

FT-IR spectra of non-treated and pretreated coconut fiber samples were obtained using the spectrophotometer FT-IR model Spectrum Two (PerkinElmer, EUA) with quartz surface, in the frequency region of 4000 –650 cm^–1^ with Horizontal Attenuated Total Reflectance (HATR). For IL, samples from each pretreatment step were combined with dry potassium bromide (KBr). This blend was then compacted into a disc shape using a manual press designed to produce KBr disks. Each disk was placed in the support of the FT-IT spectrophotometer, Model Spectrum Two (PerkinElmer, EUA), and scanned in the wavenumber region of 4000–400 cm^–1^ with a resolution of 4 cm^–1^ and 32 scans per min.

#### Scanning electron microscope (SEM) analysis

The morphology of the fibers was assessed by placing each sample on a support with a carbon fiber, then metalizing it with silver to enhance conductivity and obtaining high-quality images. The images were observed using a Scanning Electron Microscope, Model TM3000 (Hitachi, Tokyo, Japan).

#### Thermogravimetric analysis (TGA)

The thermal stability of samples previously dried in an oven for 24 h at 50 °C was evaluated by thermogravimetric analysis. The TGA-50 thermogravimeter (Shimadzu Corporation, Kyoto, Japan) was operated in a nitrogen atmosphere, heating from room temperature to 1000 °C at a rate of 10 °C/min.

### Statistical analysis

Statistical analyses were conducted using data from triplicate measurements. The differences among pretreatments were evaluated through a one-way analysis of variance (ANOVA), followed by Tukey’s multiple-comparison test. Statistical significance was established at a threshold of 5% (*p* < 0.05).

## Results and discussion

### Chemical characterization

#### Lignin concentration

The results of the chemical characterization indicated that the AH-OG pretreatment progressively improved lignin extraction. The liquors exhibited increased levels of IL and SL following the OG process (see Table 1). AH, which only utilizes water as a solvent under subcritical conditions, allowed partial lignin solubilization, making the biomass structure more accessible. However, the next step (OG using organic solvents) was more effective at removing lignin because it can disrupt ether and ester linkages [27].

**Table 1:** Yield and lignin concentration the pretreatment liquors of coconut fiber

Pretreatments	% Process mass yield	% Lignin yield	SL	IL	TL
Autohydrolysis	85.27 ± 0.74	18.42 ± 0.60	6.38 ± 0.38	0.99 ± 0.53	7.37 ± 0.50
Organosolv	67.30 ± 0.57	85.19 ± 0.37	26.76 ± 0.31	7.31 ± 0.33	34.08 ± 0.21

Values are presented as mean ± standard deviation, calculated from triplicate measurements. Statistically significant differences between treatments were determined based on the p-value (*p* < 0.05)

SL: soluble lignin (g/L); IL: insoluble lignin (g/L); TL: total lignin (g/L)

Additionally, lignin extraction inversely correlates with the remaining solid in the biomass, reducing yields in the treated biomass (Table 1). When a significant amount of lignin is removed, the biomass structure becomes more fragile and prone to decomposition, thereby resulting in a lower yield of solid. These results highlight the importance of sequential pretreatment AH-OG. AH prepares biomass by partially removing hemicelluloses and exposing lignin, which can then be extracted more effectively through the OG process. The combined action of AH and OG is a powerful strategy for enhancing cellulose accessibility and providing higher-purity cellulose pulp (Table 2), which is crucial for efficient processes such as enzymatic hydrolysis and improving bioproducts in biorefineries [28].

The analysis of variance (ANOVA) confirmed significant statistical differences between AH and OG pretreatments for all evaluated parameters, with F-values greater than the critical F and *p* < 0.5 for mass yield, lignin yields, SL, IL, and TL (Table 1). Also, for the CF samples in Table 2, the observed parameters showed significant statistical differences among untreated, AH, OH, and bleaching conditions, confirming the influence of the different pretreatment stages on the fractionation and composition of CF.

Furthermore, the Tukey test conducted on the data presented in Table 2 corroborated the existence of statistically significant differences among the samples with regard to alpha cellulose, hemicellulose, kappa number, and Klason lignin. Nevertheless, for holocellulose, no statistical differences were observed between the untreated and AH samples under the conditions evaluated. This result indicates that AH alone did not significantly alter the holocellulose content. However, its prior application to OG enhanced the overall effectiveness of the pretreatment, as Vieira et al. [8] reported lower holocellulose and α-cellulose contents when OG was applied alone to CF under the same conditions evaluated in the present study.

**Table 2:** Chemical characterization of coconut fiber samples

Components	%Untreated*	% Autohydrolysis*	% Organosolv*	% Bleaching*
Holocellulose	57.81 ± 0.82^a^	58.37 ± 0.78 ^a^	82.71 ± 0.59^b^	79.52 ± 0.48^c^
α-Cellulose	40.01 ± 0.71^a^	47.97 ± 0.67 ^b^	76.87 ± 0.36 ^c^	78.92 ± 0.25^d^
Hemicelluloses	17.80 ± 0.74 ^a^	10.40 ± 0.52 ^b^	5.84 ± 0.36 ^c^	0.61 ± 0.19 ^d^
Kappa number	43.31 ± 1.00 ^a^	59.04 ± 1.10 ^b^	68.02 ± 1.28 ^c^	26.09 ± 0.19 ^d^
Klason lignin	5.63 ± 0.15 ^a^	7.67 ± 0.13 ^b^	8.84 ± 0.17 ^c^	3.39 ± 0.02 ^d^

Values are presented as mean ± standard deviation, calculated from triplicate measurements

*Different superscript letters within the same row denote statistically significant differences by one-way ANOVA followed by Tukey’s post-hoc test (*p* < 0.05)

The scientific literature has not reported the use of sequential pretreatments, particularly for coconut residues. However, some studies have been employing the sequential AH-OG on various lignocellulosic residues for different purposes. Pontes et al. [29] submitted a mixture of six lignocellulosic biomasses to AH-OG solely for characterization. The study found that the OG method effectively fractionated lignin from autohydrolyzed biomass, yielding high-purity pulp. The results showed a lignin yield of 48.3% when using OG with 80% ethanol at a temperature of 200 °C for 60 min, along with AH at 206 °C.

Fialho et al. [30] also submitted wheat straw, which contained 35.74% cellulose, 33.34% hemicelluloses, and 14.57% acid-insoluble lignin, to the AH-OG pretreatment, employing 195 °C for AH and 190 °C for 2 h for OG. Delignification was reported as 75.6%. Saad et al. [31] applied AH-OG pretreatment on *Ziziphus lotus* biomass, a plant of the Rhamnaceae family, composed of 21.80% hemicelluloses, 18.4% lignin, and 30.8% glucan. The process was conducted with AH at 190 °C and OG at 180 °C for 60 min. AH improved the delignification efficiency of OG by 42.7%.

When comparing lignin yields from the revised studies, it is evident that the pretreatment effects depend on the conditions used and the type of biomass, which contains varying amounts of chemical components. This study showed a lignin yield of 85.19% (Table 1), demonstrating that the AH-OG approach produces more than what has been reported in other research. This variation highlights the need to optimize pretreatment parameters for each biomass type to improve delignification efficiency.

#### Determination of lignocellulosic components

The compositional analysis of the non-treated and treated CF samples indicated a notable increase in holocellulose and α-cellulose levels throughout the pretreatment steps (Table 2). The non-treated MC comprised 40.01% α-cellulose, which improved to 47.97% following AH, 76.87% after OG treatment, and 78.92% after chemical bleaching. Hemicelluloses content, on the other hand, progressively decreased during pretreatment, reducing from 17.80% in the non-treated sample to 0.61% after the final step.

Padilha et al. [32] submitted CF to acid-catalyzed OG pretreatment with 1% H_2_SO_4_, 80% glycerol, and 19% water at 130 °C for 2 h. This process was compared to a standard acid pretreatment under identical temperature and time conditions but using only a 1% H_2_SO_4_ aqueous solution. The findings indicated that including the organic solvent resulted in higher cellulose (65.99%) and lower hemicelluloses (7.29%) and Klason lignin (28.68%) than acid pretreatment (41.50% cellulose, 11.74% hemicelluloses, and 45.51% lignin). In a study conducted by Gonçalves et al. [33], the sequential pretreatment of CF using NaClO_2_–C_2_H_4_O_2_/AH occurred at 200 °C for 50 min. After pretreatment, cellulose increased to 71.25%, while hemicelluloses and lignin decreased to 6.67% and 4.25%, respectively.

From the discussed results, it can be observed that pretreatment increases the cellulose content in the pulp by promoting the removal of hemicelluloses and lignin. However, the efficiency of cellulose content can vary depending on the method used and the conditions applied. In this study, the sequential pretreatment AH-OG resulted in a cellulose content of 76.87%, 5.84% hemicelluloses, and 8.84% Klason lignin (Table 2), demonstrating effective fractionation of CF.

The Kappa number, which reflects the residual lignin content, initially increased following the initial pretreatment. It rose from 43.32 in the untreated fiber to 59.04 after AH and to 68.02 following OG treatment. While one might expect a lower Kappa number since treatment promoted delignification, this increase can be explained by two factors: (i) The lignin in the untreated sample is embedded in the cell wall, making it less accessible on the fiber surface, resulting in a Kappa number that does not fully represent the total lignin, thus appearing lower [34]; (ii) There may have been a redistribution or reintroduction of liquor lignin back onto the pulp samples, particularly during the OG process using the ethanol-water solution, which contributed to the increased Kappa number [35].

The effect of the ethanol-water solution is attributed to the decrease in lignin solubility due to changes in temperature and concentration. During the process, lignin breaks down with temperature; once the temperature decreases, solubility in ethanol also declines. Similarly, when water is added to the liquor, it causes dilution, which reduces the ethanol concentration [35]. These changes can cause some dissolved lignin to re-precipitate and reattach to the fiber surface, thereby increasing the residual lignin content. Nevertheless, this lignin is easily removed during the bleaching stages [35, 36], as indicated by the lower Kappa number found in the bleached samples (Table 2).

Several studies also determined the kappa number of coconut mesocarp. Main et al. [37] tested MC under different experimental conditions and characterized samples pretreated using soda-anthraquinone pulping. They obtained a kappa number of 41 under the least severe conditions (18% active alkali, 150 min, 170 °C) and 21 under the most extreme conditions (22% active alkali, 150 min, and 170 °C).

Klunklin et al. [38] determined the Kappa number in immature and mature coconut fibers, which have been treated with an alkaline solution of 15% NaOH (p/v) for 3 h at 90 °C. Three bleaching treatments were evaluated: the most severe treatment, which involved three bleaching stages, resulted in a Kappa number of 14.11 for green coconut and 15.00 for mature coconut. The least severe treatment, consisting of only one bleaching step, produced Kappa numbers of 20.89 and 21.23, respectively. By considering the literature-reported values, particularly under the optimal conditions employed, the Kappa number of 26.09 obtained in this study after bleaching (Table 2) seems comparable to the results observed in other studies.

The Kappa number analysis showed elevated Klason lignin content after AH and OG pretreatments. Nonetheless, subsequent bleaching resulted in a substantial reduction of Klason lignin content to 3.39%, indicating its effective removal during the final pretreatment phase (Table 2). Overall, the sequential AH-OG treatment followed by chemical bleaching produced a cellulose-rich pulp, characterized by increased α-cellulose and reduced hemicelluloses and lignin, demonstrating its potential for CF fractionation and subsequent production of cellulose derivatives.

### CMC Synthesis

In the synthesis of CMC from cellulose pulp, the process of carboxymethylation replaces the hydroxyl groups in the glucose structure with carboxymethyl groups. This results in an increase in molar mass and the modification of certain properties, such as water solubility and viscosity [39]. To confirm the solubility of CMC obtained through the AH-OG process applied to the CF samples, an immersion test was conducted. In this test, a small quantity of CMC was placed on the water surface within a beaker. After a few minutes, the CMC exhibited a gelatinous texture and dissolved completely, contrasting with the bleached cellulose, which did not dissolve [40].

**Fig. 1:**
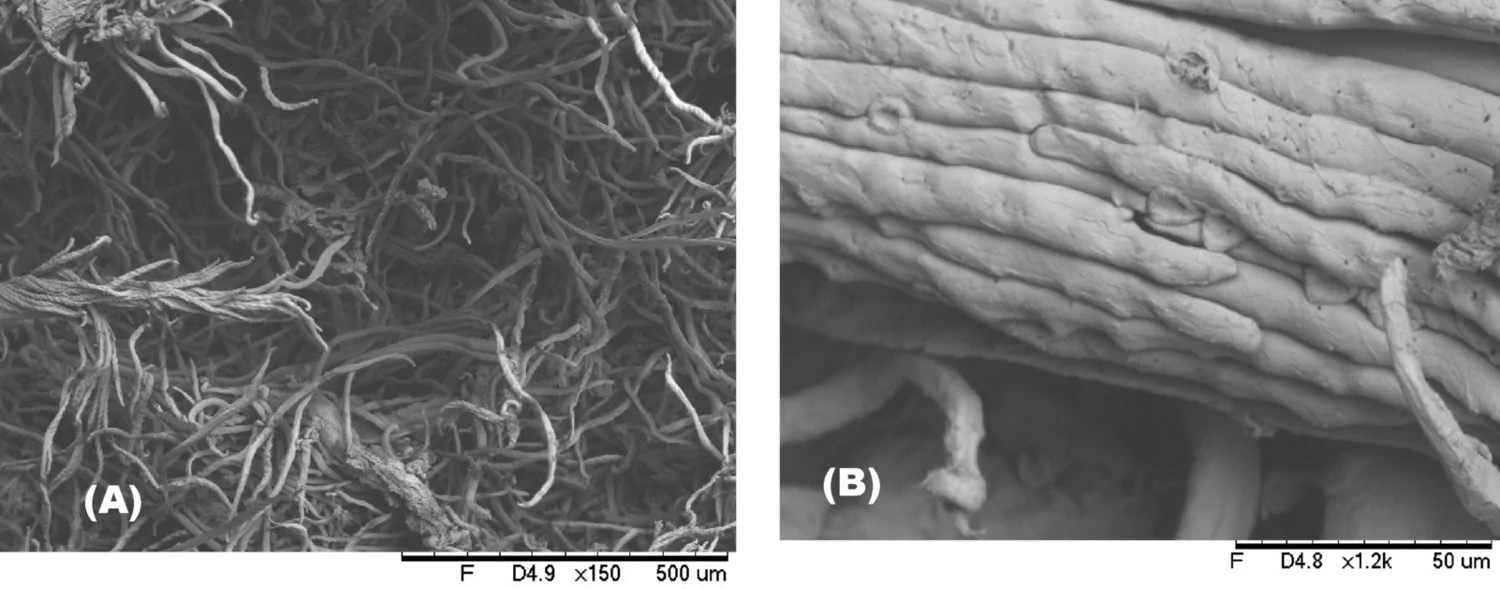
Scanning Electron Microscopy of CMC obtained by the AH-OG process in coconut fibers

**Fig. 2:**
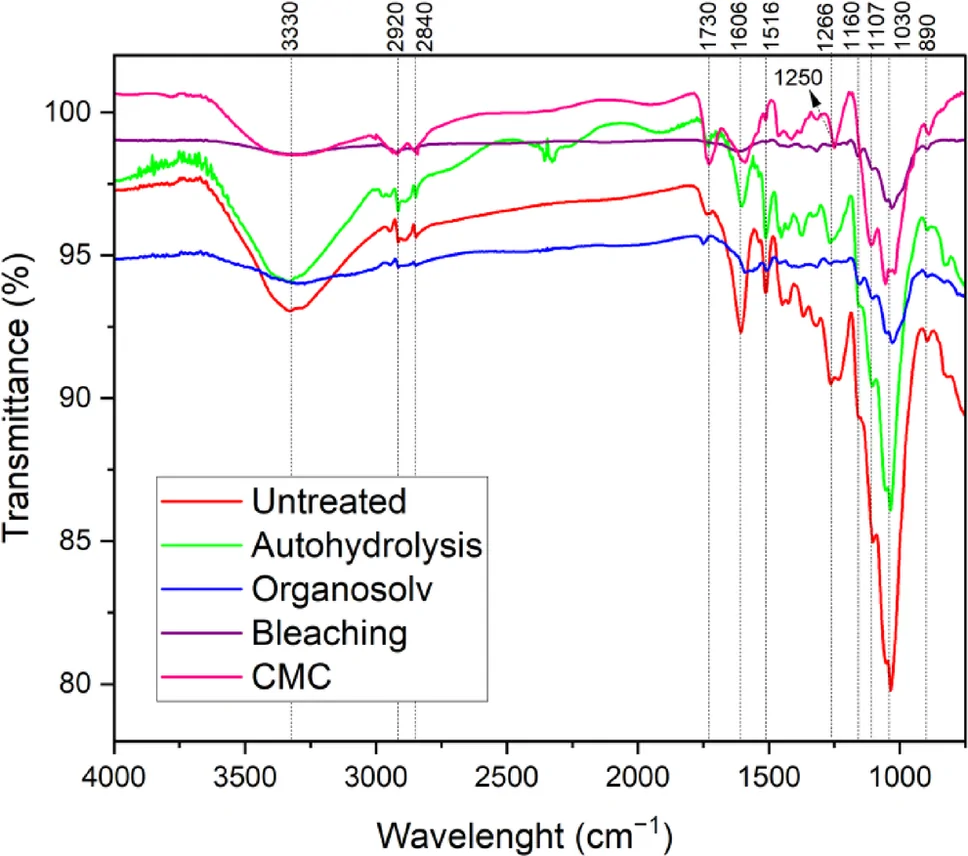
FT-IR spectra obtained by HATR of coconut fibers samples

The CMC production was confirmed by some analytical methods. The FT-IR analysis of CMC revealed peaks at 2920 and 2840 cm^−1^, indicating the addition of carboxymethyl groups to the cellulose structure, as these peaks were absent in the bleached samples [41]. Additionally, the characteristic bands of carboxylate groups were identified within peaks at 1600 cm^–1^ and 1460 cm^–1^, which are associated with the formation of the ester groups during the synthesis of CMC (Fig. 2) [42]. Figure 1 shows the SEM images of CMC, which reveal characteristics similar to the CMC obtained from other biomass sources, as described in the literature. The CMC appears as long, thin fibers, forming interwoven networks or agglomerates, with a relatively smooth surface that shows some roughness and irregularities due to the carboxymethylation process [43].

In terms of CMC production yields, the proposed pretreatment AH-OG achieved a 30.75% yield. Joyline et al. [44] reported a 39.63% yield from coconut mesocarp using alkaline pretreatment and bleaching. Togrul and Arslan [45] experimented with different conditions on beet husk to optimize the carboxymethylation process, resulting in yields that varied from 51.2% (lowest) to 68% (highest). Although CF was efficient in producing CMC, the results obtained in this study were inferior to those reported in the existing literature. One plausible explanation for these findings can be related to the type of biomass used, given that biomass varies in both composition and the structural organization of its macromolecules. Such variations can significantly influence the yield of derivatives produced. Specifically, the lower yield observed from CF compared to other types such as sugarcane bagasse, rice husk, corn straw, and banana residues may be attributed to its composition characterized by lower cellulose content and higher lignin content [46].

Besides sample type, other parameters that can influence the efficiency and yield of products, which include: (i) particle size: in this study, CF particles measured 2.5 cm, whereas other studies employed biomass samples that were milled or decreased in size to less than 1 cm, thereby increasing the contact surface with the solution and enhancing the carboxymethylation process; (ii) NaOH concentration: different concentrations of NaOH can be explored to achieve CMC with high purity and an improved yield and degree of substitution, as reported in previous studies using biomass samples such as sugarcane bagasse [47], durian rind [48], Lantana camara (an invasive weed) [49], and banana pseudostraw [50]; and (iii) optimization of operational conditions such as temperature, reaction time [51], and solvents [45].

Based on the observations, it can be concluded that to maximize the CMC yield, certain process parameters need to be modified. These modifications should aim to increase the surface contact between the sample and the solvent, as well as improve the dynamics of the reaction to facilitate the conversion process. In the case of future studies at exploring the potential of CF to produce CMS, it is recommended to reduce the size of the coconut fiber and to investigate different operational conditions in order to identify the optimal combination of time, reagent concentration, and temperature.

#### Structural characterization of samples

#### FTIR-HATR analysis

The FTIR peaks at specific wavelengths indicate alterations in the chemical composition of the samples due to the pretreatments, which allow for the identification of similarities and principal differences among the samples (Fig. 2). The absorption band at 3330 cm^−1^ is related to the stretching frequency of the –OH group, due to the content of alcohols, phenols, and carboxylic acids present in cellulose, hemicelluloses, and lignin [52]. Therefore, the presence of broad peaks at this band confirms the presence of those components in untreated and pretreated samples. Whereas the less intense peak for OG and bleached samples indicates a slight rupture of -OH linkages in cellulose and efficient extraction of lignin and hemicelluloses. Compared to the bleached sample, the CMC spectra exhibit a significant increase in the intensity of the peak at 3330 cm^−1^, which can be attributed to the additional OH stretching vibrations of the carboxymethyl groups after CMC synthesis [53].

The peaks observed at 2920 and 2840 cm^−^^1^ correspond to the stretching vibrations of -CH bonds, indicating the presence of polysaccharides and the structural components of phenylpropane, which are attributed to hemicelluloses and lignin [52]. Then, the absence of those bands was observed in the spectra of OG and bleached samples. Nonetheless, CMC spectra showed the presence of peaks in both bands due to the presence of methylene groups in the structure.

The main difference between spectra is concentrated in the fingerprint region (1800 –800 cm^–1^), where the pattern of absorption peaks is highly unique to each individual molecule, allowing for the highlighting and comparison of alterations in the molecular structure of samples [52]. In the case of CMC, the characteristic bands of the asymmetric (1600 cm^–1^) and symmetric (1460 cm^–1^) carboxylate groups (COO-) were identified in this region. Furthermore, an increase in the peaks associated with the carboxyl group at 1250 cm^−1^ and 1730 cm^−1^ was observed, corresponding to the stretching vibrations of the C–O and C = O bonds, respectively [42]. This increase is associated to the formation of ester groups during reactions.

The peaks identified at 1516, 1606, and 1266 cm^−1^ correspond to the vibration of the aromatic ring, C = C linkages, and C–O stretching vibration, respectively, which indicate the presence of lignin [54]. These peaks were prominently observed in the untreated samples and the samples subjected to AH treatment. Following OG pretreatment, chemical bleaching, and carboxymethylation, these peaks were no longer observed, indicating the removal of lignin. The bands at 1160, 1107, 1030 and 890 cm^–1^ refer to the asymmetric C–O–C stretching, asymmetric ring stretching, C-O stretching, and 1,4-β glycosidic linkages, respectively, which are related to native cellulose [54–56]. Peaks at 1160 cm^–1^ were mainly observed in the OG and bleached samples, and less pronounced in the other samples. Otherwise, peaks at 1107 cm^–1^ appeared in all samples, with higher intensity in the CMC. The peaks at 1030 cm^–1^ were also present in all samples but showed a decreasing trend: untreated > AH > CMC > bleaching > OG, which suggests alterations in the structure of native cellulose after pretreatments. The region at 890 cm^–1^ showed consistency across all samples, with minimal variation.

##### Cristinality index

Pretreatments have the potential to induce modifications in the physical, chemical, and structural characteristics of the native cellulose (known as cellulose I). Consequently, this may lead to the partial transformation of cellulose I into cellulose II (a crystalline allomorph of cellulose I) via regeneration, which may occur either when cellulose is dissolved and resolidified or through mercerization [57]. Cellulose I possesses an ordered and crystalline structure, whereas cellulose II exhibits a more amorphous configuration with reduced crystalline content, characterized by non-ordered and antiparallel cellulose chains [58]. The extent of structural modification from Cellulose I to Cellulose II can be verified using several analytical indices, including: the total crystallinity index (TCI), the lateral order index (LOI), and the hydrogen bond intensity (HBI) [59].

The TCI measures the overall proportion of crystalline material and is determined by the ratio of absorbance between the bands at 1372 cm^–1^ and 2900 cm^–1^, in which only the first band indicates crystallinity LOI indicates the level of structural perfection in crystalline regions. It is determined by the ratio of absorbances at 1430 cm^−1^ (crystalline domain) and 893 cm^−1^ (amorphous regions) [60]. HBI indicates changes in the hydrogen bond network among hydroxyl groups in cellulose, determined by the ratio of absorbance at 3400 cm^−1^ to 1320 cm^−1^ [61]. Generally, higher TCI and LOI values suggest greater crystallinity and are inversely related to HBI [62].

Table S1 presents the crystallinity index of MC samples, revealing a decreasing trend in TCI as the pretreatment steps advance: untreated > AH > OG > bleached > CMC. The progressive reduction in TCI was associated with changes in the crystalline structure of cellulose caused by the pretreatments, potentially affecting the reactivity and physical properties of the lignocellulosic material [63]. Notably, AH had the least impact on crystallinity compared to the other treatments, whereas OG and bleaching processes led to a significant reduction in crystallinity.

Regarding LOI values, different patterns were observed. The untreated sample exhibited the lowest value, while the highest was seen for the sample subjected to AH. These differences can be attributed to the effect and severity of pretreatment on the fiber structure. Milder treatments focus on partially removing lignin and hemicelluloses and have little effect on converting Cellulose I to the less ordered Cellulose II. The selective removal of amorphous components while preserving the ordered Cellulose I structure may increase the relative proportion of the crystalline fraction, resulting in higher LOI values. In contrast, more severe treatments can disrupt the native cellulose structure, resulting in decrystallization and possibly conversion to Cellulose II [63].

The pretreatments exposed more hydroxyl groups, which enhanced the formation of hydrogen bonds, as evidenced by a higher HBI. The higher HBI may therefore reflect increased hydrogen bonding associated with structural reorganization of cellulose during pretreatment [64]. As a result, a more accessible yet less organized material is obtained.

#### FT-IR analysis by KBr

**Fig. 3:**
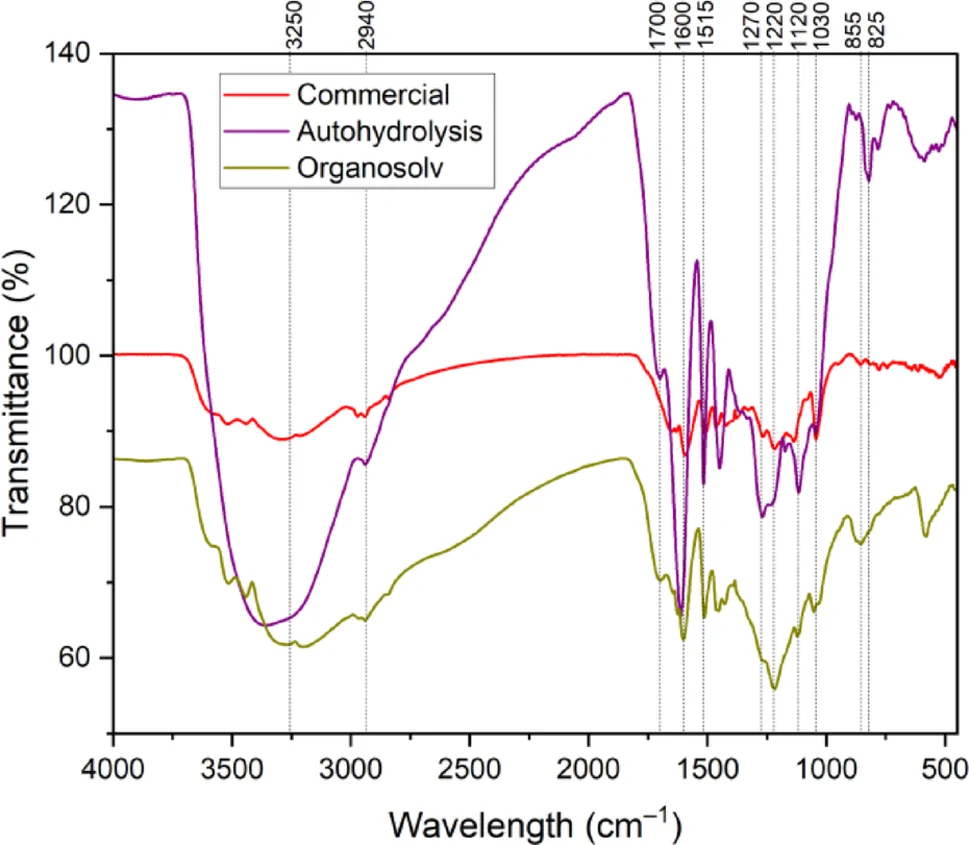
FT-IR spectra by KBr of lignins extracted from coconut fibers

The FT-IR analysis using KBr was conducted to identify the functional groups in the lignins at each pretreatment stage and to compare them with commercial lignin of alkaline origin from Sigma-Aldrich Brazil Ltda. Figure 3 shows that the lignin spectra follow the same pattern, with variations in peak intensity. OG lignin had a spectrum similar to the commercial lignin and other OG lignins found in the literature [65, 66]. Conversely, AH lignin displayed spectral variations consistent with those reported in the literature [67, 68], confirming that different chemical treatments lead to different alterations [69].

The intense peaks observed in the OG spectra are associated with the use of ethanol as the organic solvent, which facilitates the release of acetic acid from the hemicellulose structure, promoting acid hydrolysis of lignin and cleavage of its internal linkages, thereby intensifying the phenolic and carbonyl peaks in the FT-IR spectra and indicating effective lignin removal and modification [65]. Similarly, the breakdown of lignin linkages is also observed during the auto-hydrolysis process, primarily due to the dissolution of hemicelluloses and the release of organic acids [70].

The peaks associated with the vibration of the aromatic ring within the phenylpropan skeleton, corresponding to the bands at 1600 and 1515 cm^− 1^, were observed with greater intensity in the OG and AH lignin samples. Additionally, the band at 1700 cm^− 1^, which is ascribed to non-conjugated carbonyl groups, was also detected [65].

The sample spectra revealed a broad absorption band at 3250 cm^–1^, which can be attributed to aromatic and aliphatic -OH groups, and at 2940 cm^–1^, corresponding to the stretching of CH in –CH₂– and –CH₃ [71]. Peaks at 1700, 1270, 1220, 1030, and 855 cm^–1^ were observed, indicating the presence of monomeric units of guaiacyl [65, 66, 71]. Likewise, characteristic bands of the monomeric unit of syringyl were identified at 1600, 1120, and 825 cm^–1^ [66, 72]. Then, the results obtained confirm that coconut fibers are predominantly composed of lignin types, specifically guaiacyl and syringyl, a finding that is corroborated by other research studies [70].

#### SEM analysis

**Fig. 4:**
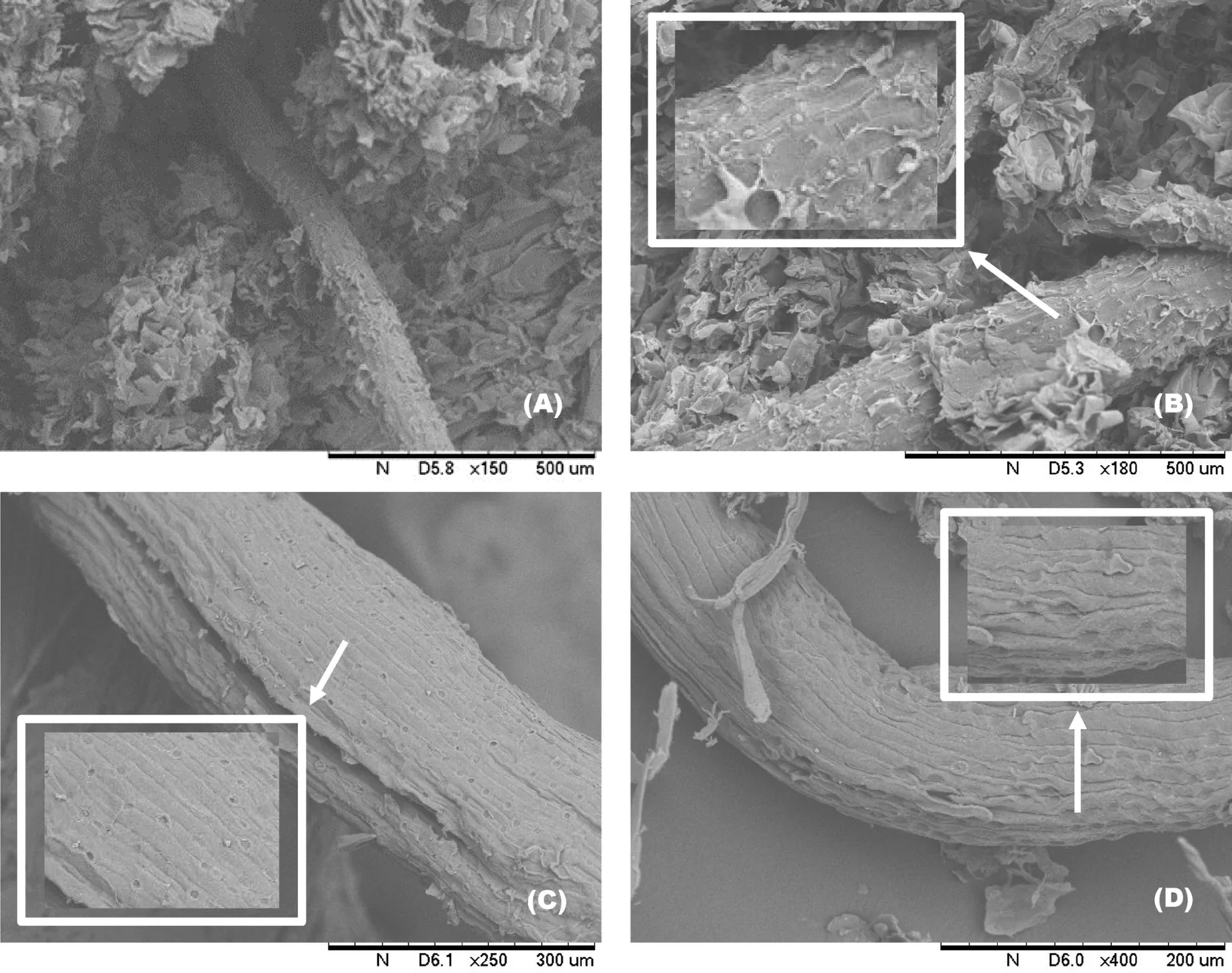
Scanning Electron Microscopy of coconut fibers: **A** untreated; **B** post-autohydrolysis; **C** post-organosolv; **D** post-bleaching

SEM analysis of CF samples (untreated, pretreated, and bleached, as present in Fig. 4 demonstrates the changes in fiber structure resulting from the pretreatment process. In Fig. 4A, the untreated CF surface exhibited a scaly layer, likely composed of pectin, waxes, and other impurities, which appeared as residual particles [38, 73]. Following pretreatment with AH, the sample retained its superficial scaly structure but began to reveal globular particles as outlined in Fig. 4B [19]. These observations suggest the partial removal of condensed products accumulated on the surface, thereby altering the fiber morphology.

OG pretreatment led to a more substantial alteration of the MC structure (Fig. 4C). The SEM image revealed a surface free of impurities, with no scorched layer present, and showed small cavities along with noticeable rugosity. These features suggest the removal of cell wall components, predominantly lignin and hemicelluloses [73]. The rugosity and cavities are more evident and pronounced in the bleached samples (Fig. 4D), which highlight the effectiveness of the chemical bleaching process in removing lignin from the fibers.

SEM images highlighted the chemical and structural changes in CF induced by the pretreatments, particularly the removal of structural components, providing insights into the resulting modifications of the cellulosic pulp. Comparable findings regarding the structure of CF were also reported by Moraes et al. [74], who utilized a NaOH alkaline solution and bleached the pulp with sodium chloride; Klunklin et al. [38], who also subjected CF to pretreatment with 15% (p/v) NaOH alkaline solution and three successive bleaching steps; Ru et al. [73] who subjected CF to alkaline pretreatment under optimal experimental conditions; and Ebrahimi et al. [75], who pretreated CF via OG using aqueous glycerol acidified as solvents.

#### TGA and DTG analysis

**Fig. 5:**
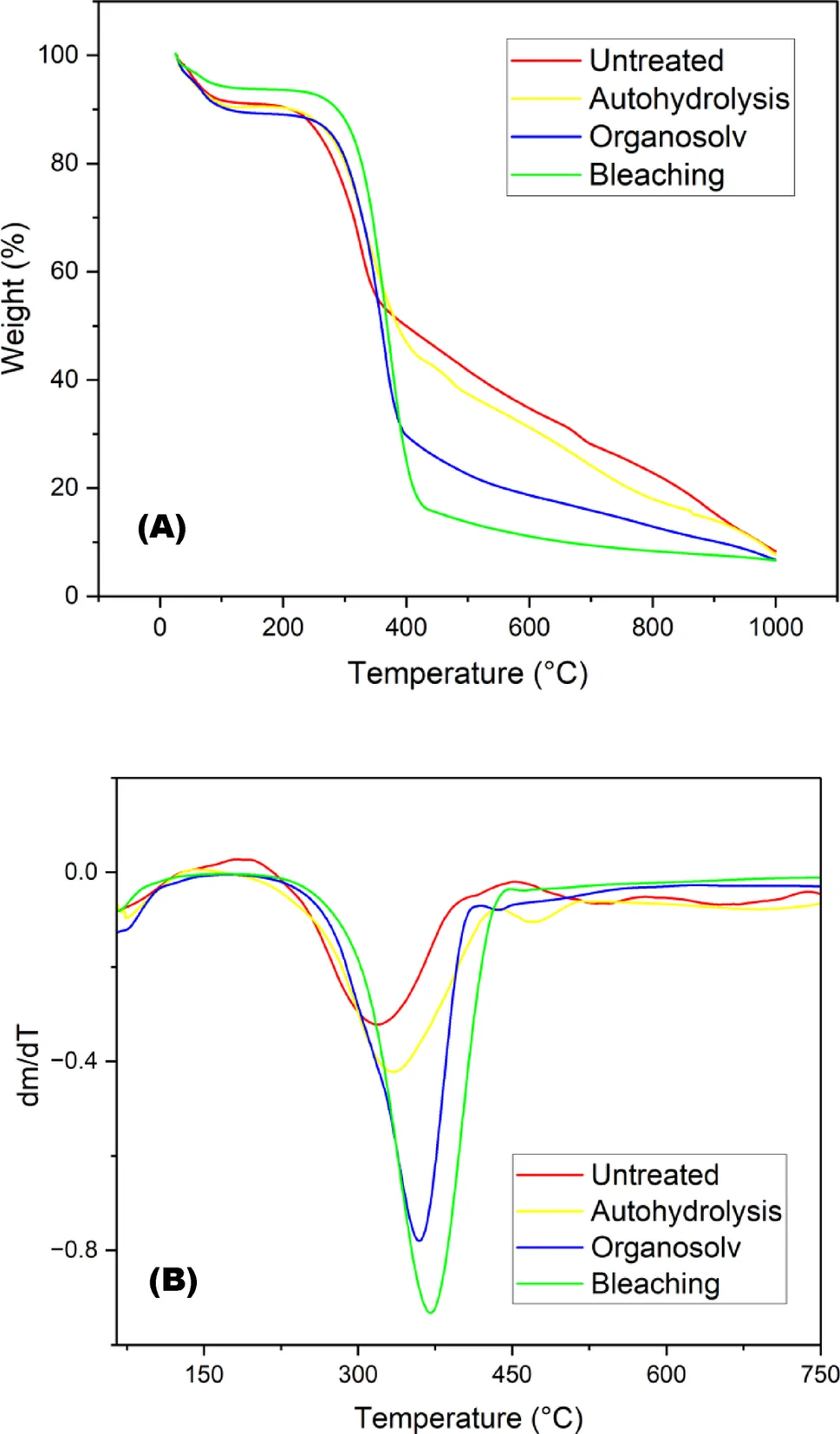
**A** TGA and **B** DTG of untreated and pre-treated coconut fibers samples

The thermogravimetric analysis of untreated and pretreated samples consistently showed a minor initial weight loss at the beginning of the curve (Fig. 5A), exhibiting comparable intensity across all samples. This initial weight loss is attributed to the evaporation of moisture and other volatile compounds at lower temperatures [76]. A more noticeable peak was seen within the temperature range of 200–400 °C (Fig. 5A). This behavior is more evident in the derivative thermogravimetry (DTG) curve, particularly for the OG and bleached samples. This temperature range indicates the degradation of the primary lignocellulosic components: cellulose, hemicelluloses, and lignin.

Hemicelluloses degradation mainly occurs between 200 °C and 315 °C. Cellulose, because of its highly ordered structure and high thermal stability, primarily degrades at 315 °C and 400 °C [77]. Lignin, on the other hand, has a complex structure made up of many aromatic rings and branches, which leads to variability in chemical linkages and causes degradation over a wide temperature range [78] that begins at 100 °C and extends up to 900 °C [77]. Thus, while hemicelluloses and cellulose are decomposing, lignin is also being partially broken down.

The TGA curves (Fig. 5A) indicate that all the samples had significant weight loss in the 200–400 °C range, which corresponds to carbohydrate degradation. OG-treated and bleached samples showed more pronounced degradation closer to 400 °C. This finding suggests a higher cellulose content in these samples, a conclusion supported by the data in Table 2. Beyond 400 °C, biomass degradation continued due to the presence of lignin, which exhibits a wide degradation range [79]. The more recalcitrant nature of these components explains the higher formation of ashes and carbonized materials at the end of the curves (near 1000 °C) for the untreated and AH samples. This observation confirms the effects of pretreatment on the removal of lignin and impurities [80].

Table S2 shows the percentage weight loss at each stage of the degradation process. The initial loss, which is more prominent in the OG samples, indicates a higher moisture content compared to the other samples. The second stage, more noticeable in the bleached samples, confirms their higher cellulose content, as previously discussed. In the temperature range associated with lignin and carbonized material decomposition (> 400 °C), the mass loss increases in the following order: untreated > AH > OG > bleaching. This pattern indicates progressive removal of hemicelluloses, lignin, and other impurities during pretreatment, resulting in an increased relative cellulose content, consistent with the data in Tables 1 and 2.

## Conclusion

The sequential AH-OG pre-treatment effectively extracted hemicelluloses and lignin, while also increasing the α-cellulose content in the biomass samples. This was confirmed by chemical analyses, which showed a residual Klason lignin of 3.39% and a Kappa number of 26.09. The enhanced cellulose accessibility was fundamental for producing CMC with a yield of 30.75%. These findings were supported by additional analyses. TGA indicated significant mass loss for cellulose, FT-IR identified pronounced cellulose peaks and the carboxymethyl group, and SEM confirmed the removal of lignin and hemicellulose and the successful production of CMC fibers. Future research should focus on optimizing sequential pretreatment conditions and the synthesis of CMC to enhance yield and properties. Additionally, it is important to evaluate the scalability of the process and its economic viability for industrial application.

## Supplementary Information

Below is the link to the electronic supplementary material.

Supplementary Material 1.

## Data Availability

No datasets were generated or analysed during the current study.
