# Supplementary material for: Sequential autohydrolysis-organosolv pretreatment for cellulose derivative production
Source: Bioprocess Biosyst Eng. 2026 Sep 19;49(11):2975–89. doi: 10.1007/s00449-026-03425-4 (PMC13661642; doi:10.1007/s00449-026-03425-4)
Supplement: Supplementary file 1 [file 449_2026_3425_MOESM1_ESM.docx]

**Sequential Autohydrolysis-Organosolv Pretreatment for Cellulose Derivative Production**

Fabrícia Vieira^1^, Hortência E. P. Santana^1^, Osiris Ashton Vital Brazil^2^, Isabelly Pereira Silva^1,3^,
Daniel Pereira Silva^1,3,4,5,6*^, Denise Santos Ruzene^1,3,4,6^

¹ RENORBIO – Northeastern Biotechnology Network, Federal University of Sergipe, São Cristóvão, SE 49107-230, Brazil

² Plural Cooperative of Work, Consulting, Research, and Services, São Paulo, SP 04013-040, Brazil

^3^ CCET – Center for Exact Sciences and Technology, Federal University of Sergipe, São Cristóvão, SE 49107-230, Brazil

^4^ PROBIO – Graduate Program in Biotechnology, Federal University of Sergipe, São Cristóvão, SE 49107-230, Brazil

^5^ PPGPI – Graduate Program in Intellectual Property Science, Federal University of Sergipe, São Cristóvão, SE 49107-230, Brazil

^6^ CISAS – Center for Research and Development in Agrifood Systems and Sustainability, Instituto Politécnico de Viana do Castelo, 4900-347, Viana do Castelo, Portugal

^*^Corresponding author at: Federal University of de Sergipe, Center for Exact Sciences and Technology, Rodovia Marechal Rondon, s/n, Jardim Rosa Elze, São Cristóvão, Sergipe 49107-230, Brazil.

E-mail address: silvadp@hotmail.com (Daniel P. Silva

**Supplementary material**

**Figures**

**Figure S1**. Sequential pre-treatment of coconut fibers for the carboxymethylcellulose (CMC) production flowchart

**Tables**

**Table S1.** Crystallinity indices of coconut fiber

| **Pretreatments** | **Index** | | |
| --- | --- | --- | --- |
|  | **TCI** | **LOI** | **HBI** |
| Untreated | 1.6206 | 0.8249 | 0.8505 |
| Autohydrolysis | 1.3924 | 0.9876 | 1.5821 |
| Organosolv | 1.0019 | 0.9142 | 1.0743 |
| Bleaching | 0.9454 | 0.9445 | 1.0227 |

TCI: Total Crystallinity Index; LOI: Lateral Order Index; HBI: Hydrogen Bond Intensity

**Table S2.** Thermal decomposition stages and main mass losses from TGA of Coconut Fiber

| **Pretreatments** | **1st wt% loss** | **2nd wt% loss** | **Residual wt% loss** |
| --- | --- | --- | --- |
|  | **27 - 120 °C** | **200 - 400 °C** | **> 400 °C** |
| Untreated | 8.72 | 40.58 | 41.56 |
| Autohydrolysis | 9.12 | 43.49 | 39.11 |
| Organosolv | 10.35 | 59.37 | 22.94 |
| Bleaching | 6.32 | 68.92 | 18.03 |
